# Resveratrol inhibits NLRP3 inflammasome activation to alleviate bovine mastitis by promoting PINK1-mediated mitophagy

**DOI:** 10.5713/ab.250935

**Published:** 2026-03-11

**Authors:** Junpeng Huang, Longwei Sun, Shujing Tan, Ran Yu, Weiguo Zhao, Chengmin Li

**Affiliations:** 1 Jiangsu Key Laboratory of Sericultural and Animal Biotechnology, School of Biotechnology, Jiangsu University of Science and Technology, Zhenjiang, China; 2 Key Laboratory of Silkworm and Mulberry Genetic Improvement, Ministry of Agriculture and Rural Affairs, The Sericultural Research Institute, Chinese Academy of Agricultural Sciences, Zhenjiang, China; 3 Livestock Industrial Development Center of Shengzhou, Shengzhou, China

**Keywords:** Mastitis, Mitophagy, NLRP3 Inflammasome, Resveratrol, Transition Dairy Cow

## Abstract

**Objective:**

Negative energy balance in transition cows elevates the circulating concentrations of non-esterified fatty acids (NEFA), potentially leading to mastitis and posing a significant threat to the dairy industry. Resveratrol is a polyphenolic compound with anti-inflammatory properties, yet its role in NEFA-induced inflammation in bovine mammary epithelial cells (BMECs) remains unclear. This study aimed how resveratrol protects against mastitis and its underlying mechanisms.

**Methods:**

BMECs were pre-treated with 100 μM resveratrol for 24 h, and then treated with 0.9 mM NEFAs for 4 h, and a PINK1 inhibitor was used to assess the mechanisms involved. Furthermore, a mouse model of mastitis was utilized to evaluate the hepatoprotective effects of resveratrol against mastitis *in vivo*.

**Results:**

Resveratrol significantly attenuated the NEFA-induced inflammatory response, evidenced by reduced levels of NLRP3 inflammasome components (NLRP3, caspase1, IL-1β) and pro-inflammatory cytokines (IL-6, IL-1β and TNF-α). Mechanistically, resveratrol promoted mitophagy by upregulating levels of LC3-II, PINK1, and Parkin, while downregulating P62 expression. The anti-inflammatory effect of resveratrol was reversed when PINK1 was inhibited. *In vivo* experiments confirmed that resveratrol alleviated mammary gland inflammation and enhanced PINK1-mediated mitophagy.

**Conclusion:**

This study demonstrates that resveratrol mitigates NLRP3-mediated inflammation by activating PINK1-mediated mitophagy, suggesting its potential as a therapeutic option for mastitis in perinatal dairy cows with negative energy balance.

## INTRODUCTION

The periparturient phase (3 weeks before and after parturition) in dairy cows is characterized by drastic physiological and metabolic changes. These changes are generally attributable to the development of inflammatory response, which negatively impact dairy cow health and productivity, posing a significant challenge to the dairy industry [1–3]. Particularly, growing evidence indicates that abrupt management changes from late dry period to early-lactation can exacerbate mammary inflammation, potentially compromising mammary function and subsequent lactation performance [4,5]. Additionally, periparturient dairy cows suffering from mastitis may experience reduced fertility and metabolic dysfunction, which can lead to a substantial increase in production costs [6].

It is currently believed that nearly all transition cows in particular undergo the state of negative energy balance (NEB), which can initiate fat mobilization and lead to a high concentration of non-esterified fatty acid (NEFA) in the blood circulation. In the meantime, the elevated circulating NEFA can be transported into mammary gland and potentially stimulate inflammatory reactions, which thereby leading to the occurrence and exacerbation of mastitis [7–9]. Nucleotide-binding oligomerisation domain-like receptor pyrin domain-containing 3 (NLRP3) inflammasome, a multiprotein complex, can be activated by NEFA and drive the upregulation of inflammatory cytokines, thus involving in the pathogenesis of inflammatory disorders [10]. In the course of NLRP3 inflammasome activation, autophagy receptor protein SQSTM1/p62 is recruited to damaged mitochondria, initiating mitophagy through the PTEN-induced putative kinase 1 (PINK1)/Parkin-dependent pathway [11,12]. Several researches have demonstrated that the enhancement of mitophagy can suppress NLRP3 inflammasome activation [13–15]. Therefore, effective approaches to mitigate inflammatory responses according to the interplay between NLRP3 activation and mitophagy may contribute to the health of transition cows.

Resveratrol (3,4’,5-trihydroxy-stilbene, RES), a well-absorbed natural polyphenolic bioactive compound found in grapes, mulberries, peanuts and some other plants, exhibits diverse biological and pharmacological activities in animals, including anti-inflammatory, antioxidant and anti-cancer [16–18]. On the other hand, resveratrol can preserve mitochondrial integrity by promoting PINK1/Parkin-dependent mitophagy [19–21]. Laboratory studies, both *in vivo* and *in vitro* evidence, demonstrated that resveratrol presented protection by activating PINK1-mediated mitophagy and inhibition of pro-inflammatory factor production [22–24]. Moreover, our previous study revealed that resveratrol could restore mitochondrial function in NEFA-stimulated bovine mammary epithelial cells (BMECs) [25]. Thus, we hypothesize that resveratrol could mediate the inflammatory response of BMECs through mitochondria pathway in response to NEFA. The objective of the present study was to clarify the underlying molecular mechanism of resveratrol regulating mastitis in transition dairy cows with NEB.

## MATERIALS AND METHODS

### Cell culture and treatments

BMEC lines were obtained from School of Biotechnology in Jiangsu University of Science and Technology. Cells were cultured to 80%–90% confluence in Dulbecco’s Modified Eagle’s Medium (DMEM)/F12 medium (Gibco) supplemented with 10% fetal bovine serum (FBS; Gibco), penicillin and streptomycin (100 U/mL; Invitrogen). The cells were maintained at 37°C in an incubator with 90% humidity and 5% CO_2_, with the medium being refreshed every 48 hours. As the experiment required, cells were pre-treated 100 μM RES for 24 h and then treated 0.9 mM NEFAs for 4 h, the preparation of NEFA and resveratrol working solutions and the treatment conditions were conducted in accordance with our previous studies [25].

### Cell transfection

Cell transfection was performed as described previously [26]. Briefly, siPINK1 and negative control siNC were designed and synthesized by GenePharma. Transfection was performed with Lipofectamine 2000 (Invitrogen), following the manufacturer’s protocol when the BMECs reached 60%–70% confluence. The PINK1 siRNA sequence is 5′-GGACUCUCU UCCUCGUCAUTT-3′, and the antisense sequence is 5′-UAUCACAAGCUUCUGCUGCTT-3′, the siNC sequence is 5′-UUCUCCGAACGUGUCACGUTT-3′, antisense sequence is 5′-ACGUGACACGUUCGGAGAATT-3′.

### Nitric oxide staining assay

The concentration of nitric oxide (NO) was determined using a commercial kit (Beiyotime) in accordance with the manufacturer’s instructions. Following treatment with NEFA and RES, the fluorescent probe DAF-FM DA was added to the BMECs, the cells were incubated at 37°C for 30 minutes and washed with PBS. Subsequently, fluorescence measurements were taken at excitation and emission wavelengths of 495 nm and 515 nm, respectively, under a fluorescence microscope (Olympus).

### Immunofluorescent staining

The cellular samples underwent fixation and permeabilization followed by blocking procedures. Then the incubation of primary antibody, rabbit anti-NLRP3 (1:200; ABclonal), was performed at 4°C overnight. Following primary antibody treatment, the cells were treated with a secondary fluorescent antibody (1:400; ABclonal) for 1 hour at room temperature. Nuclei were visualized through DAPI counterstaining. And the fluorescence images were captured by a fluorescence microscope (Olympus).

### Measurement of mitophagy flux

The treated BMECs were stained with MitoTracker Deep Red fluorescent dye (Yeasen) at 37°C for 30 minutes, after washing twice, the cells were incubated with LysoTracker Green (Yeasen) at 37°C for 30 minutes and washed twice with PBS. Fluorescent images were acquired using a fluorescence microscope (Olympus).

To quantify autophagy, we employed GFP-LC3 plasmids from our repository. Following seeding in 12-well culture plates, cellular transfections were performed prior to NEFA exposure, and green fluorescent puncta representing autophagosomes were detected by fluorescence microscope (Olympus). The cells were transfected using Lipofectamine 2000 (Invitrogenat 60%–70% confluence following the manufacturer’s protocol.

### Mitochondrial reactive oxygen species detection

Mitochondrial reactive oxygen species (ROS) levels were assessed in BMECs using the MitoSOX Red (Glpbio). Following treatment, a 5 μM staining solution was prepared and added into the cell culture dish for incubation in the dark at 37°C for 10 min. After staining, the cells were thoroughly washed three times with warm buffer. Fluorescent images were captured using a fluorescence microscopy (Olympus).

### Mitochondrial membrane potential

Mitochondrial membrane potential (ΔΨm) was evaluated using the JC-1 assay kit (Elabscience). The treated BMECs were incubated with JC-1 working solution at 37°C for 20 min under light-protected conditions. Following incubation, the cells were washed with JC-1 assay buffer. The fluorescence intensity was observed by a fluorescence microscope (Olympus) and the images was analyzed with ImageJ software (ver. 1.53t; National Institutes of Health). Each image was split into its respective red and green channels, the mean fluorescence intensity for both the red channel (J-aggregates) and the green channel (J-monomers) was measured. The ratio of red to green fluorescence intensity was calculated to assess changes in ΔΨm.

### Animal studies

To verify the therapeutic role of resveratrol on mastitis *in vivo*, a total of 40 BALB/c mice, aged 6–8 weeks and weighing 20–25 g, were procured from the Laboratory Animal Center of Jiangsu University. The mice were provided with ad libitum access to food and water and were maintained under standard housing conditions (a constant temperature of 22°C–23°C, relative humidity of 55±5%, and 12 h light/dark cycle). Following a one-week adaptation period, the mice were randomly assigned to five groups (n = 8 per group): the control group, the high-fat diet (HFD) group, the HFD+RES30 group, HFD+RES60 group, HFD+RES120 group. The HFD+RES experimental groups received a HFD supplemented with 30, 60, 120 mg/kg/day of RES, respectively. And the specific treatment procedures as described previously [25]. Following pregnancy, the female mice were housed individually and mortality was not observed in the HFD or resveratrol-treated groups during the 10-week experimental period.

Mice were subjected to a 12-hour fasting between lactation days 9 to 14, followed by euthanasia via cervical dislocation. Blood samples were obtained and centrifuged at 1,370×g for 10 minutes at 4°C, subsequently stored at −20°C until analysis. The fourth pair of mammary glands were harvested and stored at −80°C for future analysis.

### Enzyme-linked immunosorbent assay

Concentrations of proinflammatory cytokines, including tumor necrosis factor (TNF)-α, interleukin (IL)-1β and IL-6 in the cell supernatant and serum were determined using commercial enzyme-linked immunosorbent assay (ELISA) kit (MIBio) following the manufacturer’s protocol. Optical density measurements were performed using a microplate reader (BioTek).

### Real-time quantitative polymerase chain reaction analysis

RNA quality assessment was performed using a NanoDrop 1000 spectrophotometer (Thermo Fisher Scientific) to determine nucleic acid concentration and purity. DNA contamination was removed using DNase I, and cDNA was synthesized using the PrimeScript RT Master Mix kit (TaKaRa). Real-time quantitative polymerase chain reaction (PCR) was conducted on an Applied Biosystems 7500 HT system using SYBR Premix Ex Taq (TaKaRa) according to manufacturer specifications. The primer sequences were shown in Table 1. Relative gene expression levels were calculated using the 2^^−ΔΔCt^ method, with GAPDH serving as the reference gene.

### Western blotting

BMECs and mammary tissues were lysed and centrifuged at 16,000×g for 15 minutes at 4°C, and the protein concentration in the supernatant was measured using the BCA Protein Assay Kit (Beyotime). The proteins were separated by SDS-PAGE and subsequently electrotransferred onto PVDF membranes using a semi-dry transfer system (GenScript). The membranes were blocked with 5% non-fat milk in TBST at room temperature for 2 hours, and then incubated overnight at 4°C with the following primary antibodies: rabbit anti-NLRP3, P62, LC3, PINK1, Parkin, GAPDH (1:1,000; Proteintech), rabbit anti-Caspase-1 (1:2,000; Wanleibio), rabbit anti-IL-1β (1:1,000; Santa Cruz Biotechnology), TIMM23, TOM20 (1:1,000; ABclonal), rabbit anti- Tubulin (1:1,000; Bioworld). The membrane was then washed with TBST and incubated with an HRP-conjugated secondary antibody (1:5,000; Proteintech). Immunoreactive bands were visualized using enhanced chemiluminescence (ECL) reagents (Pierce) and quantified by ImageJ software (National Institutes of Health).

### Statistical analysis

All experiments were conducted with at least three biological replicates and repeated independently three times. The data were statistically analyzed using GraphPad Prism 9.5.0 software. One-way analysis of variance (ANOVA) followed by Tukey’s test was performed for multiple group comparisons, and a Student’s t-test was used for comparisons between two groups. p<0.05 was considered to be statistically significant.

## RESULT

### Resveratrol suppresses non-esterified fatty acid-induced NLRP3 inflammasome activation in bovine mammary epithelial cells

**Figure 1:**
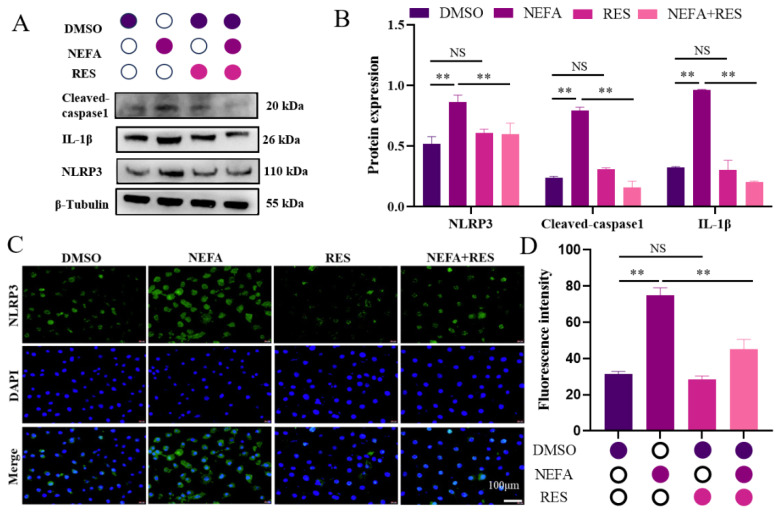
Effect of resveratrol on non-esterified fatty acids (NEFA)-induced nucleotide-binding oligomerisation domain (NOD)-like receptor pyrin domain-containing 3 (NLRP3) inflammasome activation in bovine mammary epithelial cells (BMECs). BMECs were pretreated with 100 μM resveratrol (RES) for 24 h and then stimulated with 0.9 mM NEFA for 4 h. (A) Protein expression of NLRP3 inflammasome-related genes in BMECs detected by Western blotting. (B) Ratios of NLRP3, caspase1 and IL-1β to β-Tubulin. (C, D) Immunofluorescence staining of NLRP3 in BMECs. The green fluorescence visualized NLRP3, DAPI was used to visualize the nuclei (blue). Scale bar: 100 μm. Data are presented as mean± standard error of the mean (SEM) of three independent experiments. NS indicates p>0.05, ** p<0.01. IL, interleukin.

Given the pro-inflammatory nature of NLRP3 inflammasome signaling and anti-inflammatory activity of resveratrol, the regulation of resveratrol on NLRP3 inflammasome activation was assessed. As illustrated in Figures 1A, 1B, the results showed that compared to the DMSO group, NEFA significantly elevated the expression of NLRP3 inflammasome-associated components, including NLRP 3, caspase1 and IL-1β. Conversely, compared with the NEFA group, the NLRP3 inflammasome-related proteins were remarkably reduced following pretreatment with resveratrol. Meanwhile, immunofluorescence analysis of BMECs using anti-NLRP3 antibody further demonstrating that NEFA treatment enhanced NLRP3 expression while resveratrol attenuated its induction (Figures 1C, 1D). The results indicated that resveratrol inhibits the activation of NLRP3 inflammasome signaling.

### Resveratrol inhibits non-esterified fatty acid-induced inflammatory factor expression in bovine mammary epithelial cells

**Figure 2:**
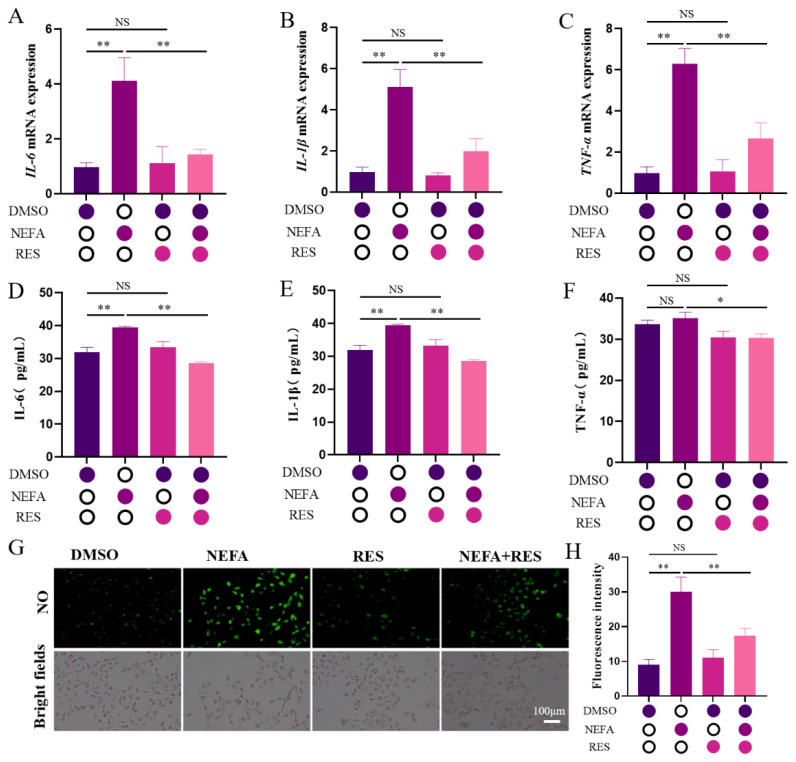
Effect of resveratrol on NEFA-induced inflammatory factor expression in BMECs. (A–F) mRNA and protein expressions of interleukin (IL)-6, IL-1β and tumor necrosis factor (TNF)-α determined by RT-qPCR and ELISA, respectively. (G, H) Fluorescence staining of nitric oxide (NO) in BMECs. Scale bar: 100 μm. Data are presented as mean±SEM of three independent experiments. NS indicates p>0.05, * p<0.05, ** p<0.01. NEFA, non-esterified fatty acid; BMEC, bovine mammary epithelial cell; RT-qPCR, real-time quantitative polymerase chain reaction; ELISA, enzyme-linked immunosorbent assay; SEM, standard error of the mean.

The activation of NLRP3 inflammasome can trigger the secretion of pro-inflammatory cytokines, therefore, effect of resveratrol on the release of inflammatory factors was further investigated. Compared to the DMSO group, the gene and protein expression levels of IL-6, IL-1β and TNF-α in the NEFA-stimulated BMECs significantly increased. While after resveratrol treatment, the production of pro-inflammatory cytokines was significantly inhibited (Figures 2A–2F), indicating that resveratrol could effectively suppress NLRP3 inflammasome-mediated inflammatory responses caused by NEFA. Consistent with these findings, NEFA administration markedly elevated the levels of the inflammatory mediator NO and pretreatment with resveratrol reversed this behavior (Figures 2G, 2H).

### Resveratrol promotes mitophagy in non-esterified fatty acid-treated bovine mammary epithelial cells

**Figure 3:**
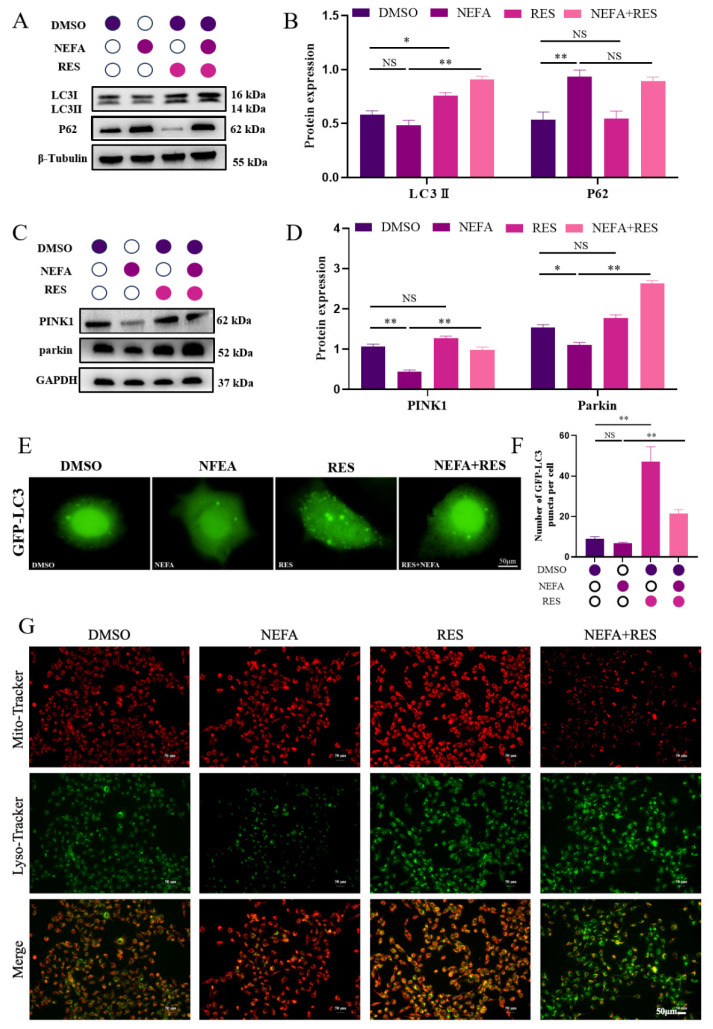
Effect of resveratrol on mitophagy in NEFA-treated BMECs. (A) Protein expression of LC3 and P62 in BMECs detected by Western blotting. (B) Ratios of LC3II and P62 to β-Tubulin. (C) Protein expression of PINK1 and Parkin in BMECs detected by Western blotting. (D) Ratios of PINK1 and Parkin to GAPDH. (E) Autophagosome formation was assessed using a GFP-LC3 reporter plasmid, green fluorescent puncta indicate autophagosomes. Scale bar: 50 μm. (F) Quantification of the number of GFP-LC3 puncta per cell. (G) Mitophagy flux was evaluated by co-staining with MitoTracker Red (red) and LysoTracker (green). Yellow signals in merged images indicate colocalization of mitochondria and lysosomes, reflecting mitophagy. Scale bar: 50 μm. Data are presented as mean±SEM of three independent experiments. NS indicates p>0.05, * p<0.05, ** p<0.01. NEFA, non-esterified fatty acid; BMEC, bovine mammary epithelial cell; SEM, standard error of the mean.

The activation of NLRP3 inflammasome is initiated primarily by impaired mitochondrial function, mitophagy is therefore an important regulator of NLRP3 activation as it removes damaged and dysfunctional mitochondria [27]. In our study, a significant reduction in mitophagy was observed in NEFA-treated BMECs, as evidenced by the decreased protein expression of mitophagic markers LC3-II, PINK1, Parkin and increased expression of selective autophagy receptor P62. On the contrary, pretreatment with resveratrol significantly upregulated the expression of LC3-II, PINK1, Parkin and downregulated P62 expression level (Figures 3A–3D). At the same time, quantitative analysis of GFP-LC3 puncta revealed reduced autophagosome formation (green puncta) following NEFA treatment, while resveratrol pretreatment reversed this effect (Figures 3E, 3F). The colocalization of lysosomes and mitochondria was significantly increased in the resveratrol treatment group, implying that resveratrol enhanced the lysosome-mitochondria fusion processing (Figure 3G). Collectively, these results indicated that resveratrol effectively enhanced mitophagy in NEFA-treated BMECs.

### Knockdown of PINK1 blocks mitophagy in non-esterified fatty acid-treated bovine mammary epithelial cells

**Figure 4:**
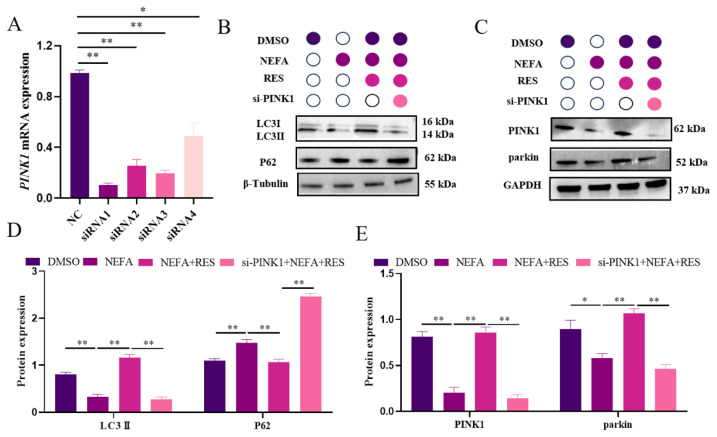
Effect of PINK1 silencing on mitophagy in NEFA-treated BMECs. (A) PINK1 gene silencing efficiency in BMECs determined by RT-qPCR. (B, C) Protein expression of mitophagic markers (LC3, P62, PINK1 and Parkin) in BMECs detected by Western blotting under different treatments: DMSO (control), NEFA (0.9 mM, 4 h), RES (100 μM, 24 h), si-PINK1 (PINK1 siRNA transfection). (D) Ratios of LC3II and P62 to β-Tubulin. (E) Ratios of PINK1 and Parkin to GAPDH. Data are presented as mean±SEM of three independent experiments. NS indicates p>0.05, * p<0.05, ** p<0.01. NEFA, non-esterified fatty acid; BMEC, bovine mammary epithelial cell; RT-qPCR, real-time quantitative polymerase chain reaction; SEM, standard error of the mean.

Mitophagy is initiated by PINK1-mediated phosphorylation of ubiquitin, which subsequently activates Parkin [28]. The expression of PINK1 was silenced to further uncover the underlying mechanisms of resveratrol regulating mitophagy in BMECs. As shown in Figure 4A, PINK1 was effectively knocked down, which satisfied the requirements for the follow-up experiments. Furthermore, knockdown of PINK1 markedly reverted the improve effect of resveratrol on mitophagy in NEFA-stimulated BMECs, as indicated by downregulation of LC3-II, PINK1, Parkin and upregulation of P62 (Figures 4B–4E).

### Inhibiting PINK1-medicated mitophagy prevented the protective effect of resveratrol on mitochondrial damage in non-esterified fatty acid-induced bovine mammary epithelial cells

**Figure 5:**
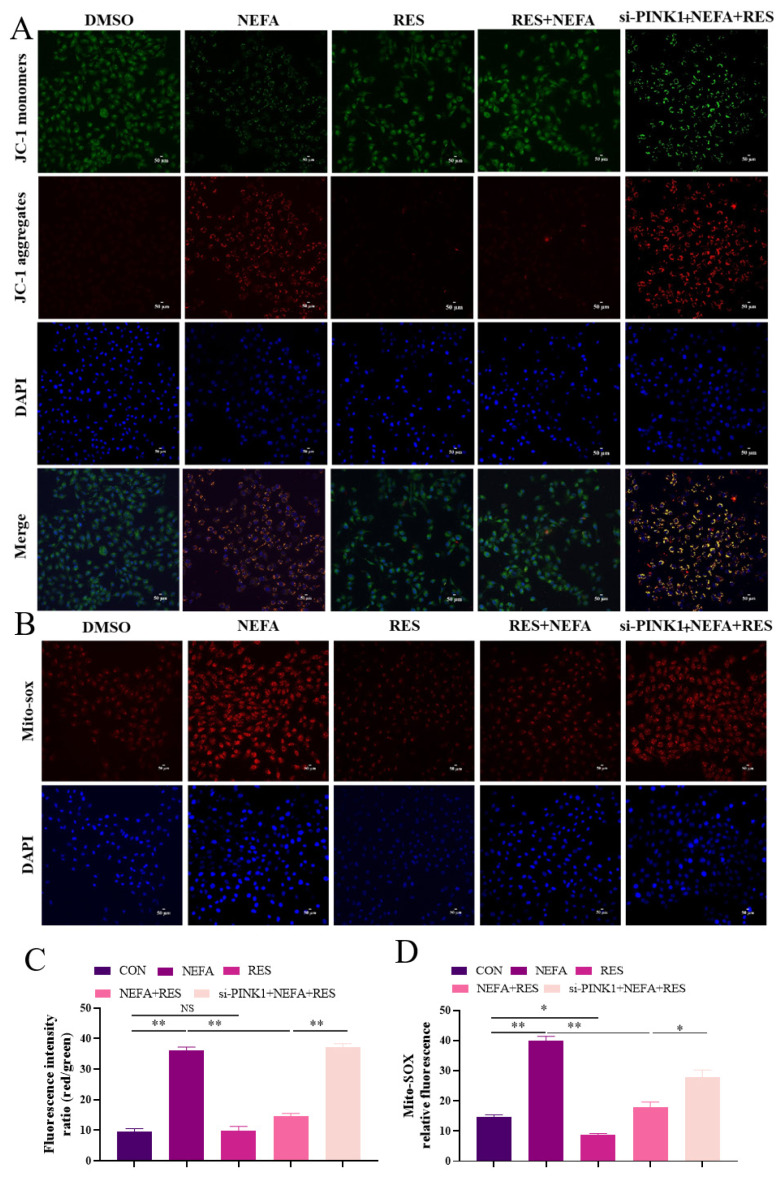
Effect of mitophagy inhibiting on mitochondrial damage in NEFA-treated BMECs. Cells were transfected with si-PINK1 and then treated as indicated. (A, C) Assessment of mitochondrial membrane potential via JC-1 staining under different treatments: DMSO, NEFA, RES, NEFA+RES and si-PINK1+NEFA+RES. Green and red fluorescence represent monomeric and aggregated forms of JC-1, respectively. Scale bar: 50 μm. (B, D) Mitochondrial ROS levels under different treatments were detected using the MitoSOX Red fluorescent staining. Scale bar: 50 μm. Data are presented as mean±SEM of three independent experiments. NS indicates p>0.05, * p<0.05, ** p<0.01. NEFA, non-esterified fatty acid; BMEC, bovine mammary epithelial cell; ROS, reactive oxygen species; SEM, standard error of the mean.

Considering that mitophagy negatively regulates NLRP3 inflammasome activation by selectively eliminating damaged mitochondria, we further investigated the impact of resveratrol on NEFA-induced mitochondrial damage. As illustrated in Figures 5A, 5B, resveratrol treatment effectively alleviated the NEFA-induced mitochondrial membrane potential loss and ROS accumulation in BMECs, whereas inhibiting mitophagy by knocking down PINK1 counteracted the protective effects of RES on mitochondria.

### Inhibiting PINK1-medicated mitophagy reverses the anti-inflammatory effect of resveratrol on non-esterified fatty acid-induced bovine mammary epithelial cells

**Figure 6:**
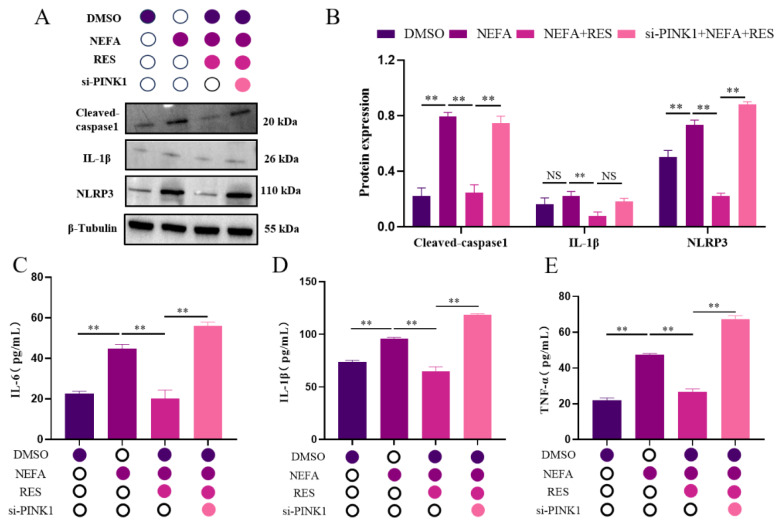
Effect of mitophagy inhibiting on inflammatory factors expression in NEFA-treated BMECs. (A) Protein expression of NLRP3 inflammasome-related genes detected by Western blotting under different treatments: DMSO, NEFA, NEFA+RES, NEFA+RES+si-PINK1. (B) Ratios of Cleaved-caspase1, IL-1β and NLRP3 to β-Tubulin. (C–E) Levels of IL-6, IL-1β and TNF-α determined by ELISA under different treatments. Data are presented as mean±SEM of three independent experiments. NS indicates p>0.05, ** p<0.01. NEFA, non-esterified fatty acid; BMEC, bovine mammary epithelial cell; IL, interleukin; TNF, tumor necrosis factor; ELISA, enzyme-linked immunosorbent assay; SEM, standard error of the mean.

We then explored whether resveratrol alleviates NEFA-induced inflammation in BMECs through PINK1-mediated mitophagy. As shown in Figure 6, blocking PINK1-medicated mitophagy counteracts the suppressive effect of resveratrol on inflammatory factors expression in BMECs, as demonstrated by the enhanced activation of NLRP3 inflammasome signaling and increased expression of IL-6, IL-1β and TNF-α. Collectively, these findings suggested that resveratrol suppressed the activation of the NLRP3 inflammasome by promoting mitophagy.

### Resveratrol promotes mitophagy in the mammary gland of high-fat diet mice

**Figure 7:**
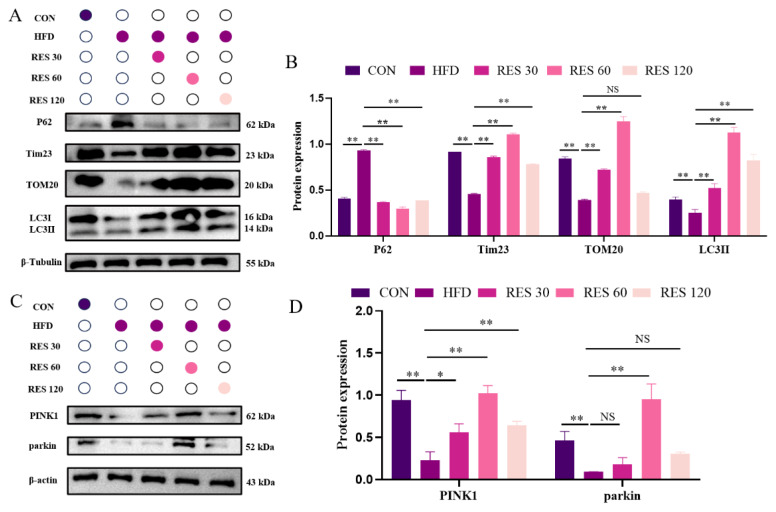
Effect of resveratrol on mitophagy in the mammary gland of high-fat diet (HFD) mice. Female BALB/c mice were fed a normal diet (CON) or a HFD supplemented with resveratrol at 30, 60, or 120 mg/kg/day for 10 weeks. (A) Protein expression of P62, Tim 23, Tom 20 and LC3 detected by Western blotting. (B) Ratios of P62, Tim 23, Tom 20 and LC3 to β-Tubulin. (C) Protein expression of PINK1 and Parkin detected by Western blotting. (D) Ratios of PINK1 and Parkin to β-actin. Data are presented as mean±SEM (n = 8). NS indicates p>0.05, * p<0.05, ** p<0.01. SEM, standard error of the mean.

To elucidate the role of resveratrol in mitigating inflammation, we also assessed mitophagic activity in HFD mice. Results showed that HFD treatment significantly decreased mitophagic markers LC3-II, Time23, Tom20, PINK1, Parkin and increased P62 expression. Conversely, the RES+HFD group showed markedly increased mitophagic activity in the mice mammary gland, and the 60 mg/kg/day dose demonstrated the best effects (Figures 7A–7D).

### Resveratrol alleviates high-fat diet-induced mouse mastitis

**Figure 8:**
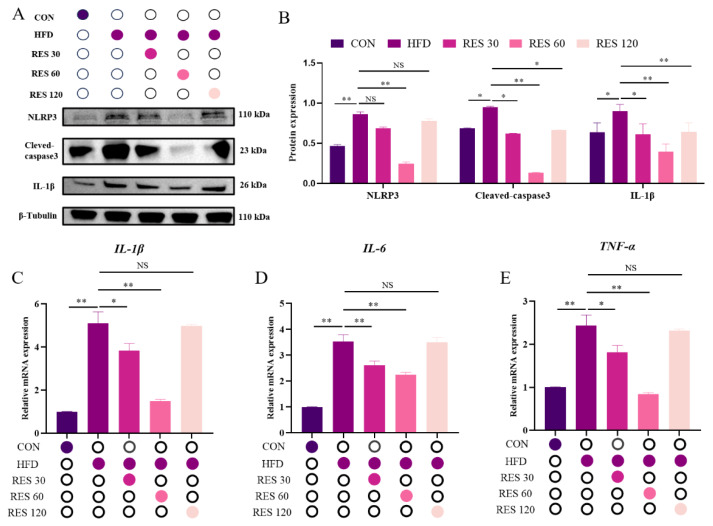
Effect of resveratrol on high-fat diet (HFD)-induced inflammatory factor expression in mammary gland of mice. (A) Protein expression of NLRP3 inflammasome-related genes detected by Western blotting under different treatments: Con (control), HFD, RES30 (30 mg/kg resveratrol supplement), RES60 (60 mg/kg resveratrol supplement), RES120 (120 mg/kg resveratrol supplement). (B) Ratios of NLRP3, Cleaved-caspase1 and IL-1β to β-Tubulin. (C–E) mRNA expressions of IL-6, IL-1β and TNF-α determined by RT-qPCR under different treatments. Data are presented as mean±SEM (n = 8). NS indicates p>0.05, * p<0.05, ** p<0. 01. IL, interleukin; TNF, tumor necrosis factor; RT-qPCR, real-time quantitative polymerase chain reaction; SEM, standard error of the mean.

The anti-inflammatory effects of resveratrol were deeply investigated in mammary gland of HFD mice. Compared to the control group, the mammary tissue of mice in the HFD group suffered a pronounced inflammatory response, as illustrated by the increased levels of NLRP3 inflammasome-related proteins NLRP3, caspase1 and IL-1β, as well as the cytokines IL-6, IL-1β and TNF-α. Nevertheless, orally administration of 30 and 60 mg/kg/day of resveratrol remarkably suppressed HFD-induced NLRP3 inflammasome activation and cytokine release in mice (Figures 8A–8E). These changes were consistent with the observed results in BMECs and indicated that resveratrol could effectively alleviate HFD-induced mouse mastitis.

## DISCUSSION

**Figure 9:**
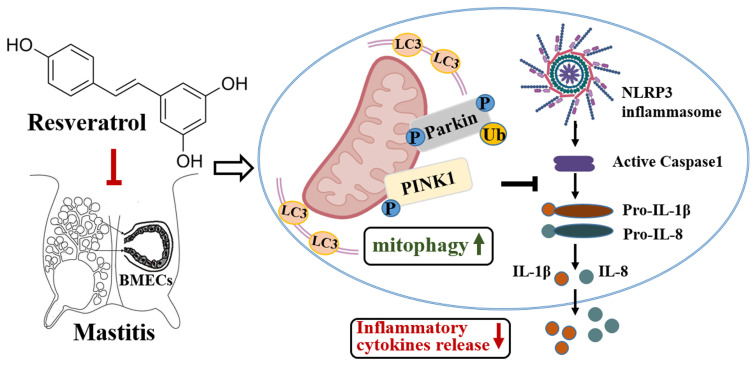
Schematic diagram of the mechanisms through which resveratrol treatment inhibits NLRP3 inflammasome activation to alleviate bovine mastitis. Resveratrol is a polyphenolic bioactive compound that exerts its anti-inflammatory activity by promoting PINK1-mediated mitophagy to blocks NLRP3 inflammasome cascade activation, ultimately decreasing cytokine release and mitigating inflammatory responses.

Dairy cow mastitis represents the most significant pathological challenge during the transition period from late gestation to early lactation. The present work elucidates the regulatory function of resveratrol in NEFA-stimulated BMECs based on its anti-inflammatory properties. Our findings revealed that resveratrol exhibited strong inhibition on NLRP3 inflammasome activation and inflammatory factor expression. From a mechanistic perspective, the present study showed that resveratrol enhanced the clearance of impaired mitochondria via the PINK-mediated mitophagy pathway, thereby attenuating NLRP3 inflammasome formation, suggesting a potential role in inflammation regulation. Additionally, animal studies using a murine model of HFD-induced inflammation revealed that resveratrol treatment attenuated the inflammatory response of mouse mammary tissues, and improved levels of mitophagy. Collectively, we validated the protective effect of resveratrol on inflammatory response both *in vivo* and *in vitro* experiments, suggesting that resveratrol may represent a novel therapeutic target for mitigating bovine mastitis in transition dairy cows with NEB (Figure 9).

During the periparturient period, dairy cows experience significant metabolic and immunological challenges. Elevated levels of NEFA, mobilized in response to NEB, play a central role in the pathogenesis of inflammation [1]. If the inflammation develops into a pathological condition, it will increase the incidence of mastitis, thereby leading to a decline in milk yield and milk quality [29]. Previous research by Contreras et al. demonstrated that NEFA supplementation promoted the levels of inflammatory mediators, including cytokines and cell adhesion molecules in bovine endothelial cells [30]. Shi et al [31] found that NEFA can act as inflammatory mediators, which initiated inflammatory dairy cows and caused injury of hepatocytes in calves. Furthermore, our previous transcriptome study revealed that excessive NEFA triggered inflammatory responses in BMECs [9]. Consistent with these previous studies, this work showed that exogenous NEFA enhanced the activation of NLRP3 signaling and release of pro-inflammatory cytokines TNF-α, IL-6, and IL-1β. All the above findings indicated that high NEFA levels could provoke inflammation in multiple cell types of transition cows, targeting the activation of the NLRP3 inflammasome represents an effective strategy for the management of inflammatory disorders.

*In vitro* and *in vivo* studies have demonstrated that resveratrol exerts its anti-inflammatory effects primarily through the suppression of inflammatory factor production. Previous research on the cytoprotective role of resveratrol in fumonisin B1-induced porcine intestinal epithelial cells showed that resveratrol effectively downregulated the expression of TNF-α, IL-1β, and IL-6 [32]. Other studies have shown that resveratrol suppresses the activation of NLRP3 inflammasome *in vitro* cell assays and *in vivo* applications [33–35]. Once activation, the NLRP3 inflammasome initiates downstream signaling cascades. Specifically, Caspase1 was recruited and then mediated the cleavage of cytokines pro-IL-1β and pro-IL-18 into their respective active forms [36]. Consistent with the above findings, our results showed that resveratrol treatment blocked the NLRP3 inflammasome cascades, reduced the expression of proinflammatory cytokines induced by NEFA in BMECs, thus uncovering the anti-inflammatory protective effect of resveratrol on mastitis of perinatal cows with NEB.

Mitochondrial impairment represents a key upstream trigger for NLRP3 inflammasome activation, and mitophagy mitigates this activation by eliminating impaired and nonfunctional mitochondria. Studies indicate that resveratrol regulates mitochondrial function, and can also mitigate stress-induced mitochondrial dysfunction [37]. Given this, we propose the hypothesis that resveratrol alleviates NEFA-induced inflammatory responses in BMECs via the mitochondria pathway. The outcomes of this study revealed that mitochondrial dysfunction induced by NEFA could be mitigated to a certain extent by resveratrol treatment. Moreover, resveratrol has been found to promote mitophagy by regulating the expression of PINK1, Parkin, LC3-II and P62, which are consistent with previous studies that resveratrol increased LPS-induced downregulation of mitophagy-related indicators [23].

During mitophagy, PINK1 cooperates with Parkin to mediate the lysosomal degradation of damaged mitochondria, which represents a well-characterized signaling cascade that activates mitophagy [38]. To further elucidate the protective mechanism of resveratrol against inflammatory response, particularly its association with PINK1/Parkin-mediated mitophagy, we knocked down PINK1 expression by transfecting with PINK-siRNA in BMECs. The results indicated that PINK1 silencing led to an increase of NLRP3 and caspase-1, as well as the pro-inflammatory cytokines IL-1β, IL-6 and TNFα expression levels. These results align with the study by Liu et al [39] and Li et al [40], which indicates that si-PINK1 reversed the inhibitory effects of resveratrol on NEFA-induced inflammatory response. This elucidated the mechanism by which resveratrol treatment modulates NLRP3 inflammasome activation through mitochondria-derived intrinsic pathway.

Transition cows experiencing NEB undergo complex metabolic changes characterized by extensive lipid mobilization. Currently, no suitable *in vivo* system adequately recapitulates this physiological state. Given ethical and clinical considerations and conserved metabolic pathways between bovines and rodents, we employed a HFD mouse model to investigate resveratrol’s anti-inflammatory properties for *in vivo* studies. The present study revealed that resveratrol exerted protective effects against HFD-induced mastitis by suppressing NLRP3 inflammasome cascade activation, which was consistent with the previous research on the anti-inflammatory role of resveratrol in adipose tissue of HFD-induced obese mice [41]. Particularly, resveratrol was found to simultaneously promote the PINK1-mediated mitophagy in the mammary gland of HFD mice. These observations are consistent with the previous work by Fan et al., which demonstrated that resveratrol treatment led to a marked enhancement in mitophagic flux [23,34].

## CONCLUSION

Collectively, this study revealed that resveratrol treatment significantly suppressed NLRP3 inflammasome-mediated inflammatory reactions in both cellular and animal models. Mechanistically, our investigations found that resveratrol exerts its anti-inflammatory activity by promoting PINK1-mediated mitophagy to blocks NLRP3 inflammasome cascade activation, ultimately decreasing cytokine release and mitigating inflammatory responses. These findings suggest that resveratrol may serve as a therapeutic alternative for mastitis in dairy cows undergo NEB.

## Figures and Tables

**Table 1:** Primers sequences for real-time quantitative PCR analysis

Gene	Accession number	Primer sequences (5′–3′)	Fragment size (bp)
*TNF-α*	NM_173966.3	F: CGACATCAACTCTCCGGGGC	198
		R: GCAATGCGGCTGATGGTGTG	
*IL-6*	NM_173923.2	F: CGCTTCACAAGCGCCTTCAC	249
		R: TGCCAGTGTCTCCTTGCTGC	
*IL-1β*	NM_174093.1	F: ACAGCCATGGCAACCGTACC	219
		R: CATGGCCACGATGACCGACA	
*β-Actin*	NM_173979.3	F: GGGCAGGTCATCACCATCGG	244
		R: TCATTGTGCTGGGTGCCAGG	
*GAPDH*	NM_001034034.2	F: CATGACCACTTTGGCATCGT	133
		R: CCATCCACAGTCTTCTGGGT	

PCR, polymerase chain reaction.

## Data Availability

Upon reasonable request, the datasets of this study can be available from the corresponding author.
